# The First Autopsy Case of Fatal Acute Cardiac Failure after Administration of Carfilzomib in a Patient with Multiple Myeloma

**DOI:** 10.1155/2019/1816287

**Published:** 2019-04-28

**Authors:** Teruhito Takakuwa, Ippei Otomaru, Taku Araki, Akiko Miura, Yotaro Fujitani, Yasuhide Mochizuki, Yoshimi Miyagi, Hideto Senzaki, Ryosuke Yamamura

**Affiliations:** ^1^Department of Hematology, Osaka Saiseikai Nakatsu Hospital, Osaka, Japan; ^2^Department of Cardiology, Osaka Saiseikai Nakatsu Hospital, Osaka, Japan; ^3^Department of Pathology, Osaka Saiseikai Nakatsu Hospital, Osaka, Japan

## Abstract

Carfilzomib (CFZ) improves progression-free survival for patients with relapsed or refractory multiple myeloma (MM) but has shown higher frequency of cardiovascular adverse events (CVAEs) than other proteasome inhibitors. We report the first autopsy case of acute death from cardiac failure shortly after administration of carfilzomib. A 74-year-old female was diagnosed with IgA MM after a 2-year period of smoldering MM. She was refractory to both bortezomib plus dexamethasone and lenalidomide plus dexamethasone therapies, so she subsequently received CFZ in combination with lenalidomide and dexamethasone. The day after the start of the therapy, she complained of severe dyspnea with a significant decline in left ventricular ejection fraction. Her acute cardiac failure rapidly progressed, and she died on day 7 of the start of CFZ. The autopsy showed invasion of inflammatory cells between the myocardial cells and very little myocardial necrosis. There was no obvious thrombus in the coronary artery of the heart, and no infarction or amyloid deposition was observed in the myocardium. Pathological findings of hypersensitivity myocarditis, a drug-induced cardiomyopathy, appeared to agree with this case except for absence of an eosinophilic infiltration of the myocardium. A CFZ-induced CVAE is generally considered reversible. However, rapidly progressing fatal heart failure like in our case is rare. To characterize CFZ-associated CVAE, further case collection is needed.

## 1. Introduction

Over the past 10 years, new drugs have progressively become clinically applicable for the treatment of multiple myeloma (MM) [1]; thus, treatment strategies for MM have undergone a considerable change. Carfilzomib (CFZ) is a second-generation proteasome inhibitor (PI) that shows excellent outcomes for relapsed and/or refractory MM. In the ASPIRE study [2], using the lenalidomide (LEN) and dexamethasone (DEX) combination therapy in the control group, overall survival was significantly prolonged by adding CFZ to the therapy. Furthermore, in the ENDEAVOR study [3], the CFZ + DEX combination therapy significantly prolonged progression-free survival compared with the bortezomib (BOR) and DEX combination therapy. However, it has been found that CFZ increases the risk of cardiovascular events. In therapies containing CFZ, the abovementioned ASPIRE study showed heart failure in 6.4% of participants, whereas the ENDEAVOR study showed congestive heart failure in 10.8% of participants, both of which were higher than that in the control group. One of our patients died of severe cardiac hypofunction following CFZ administration, after which we performed pathological autopsy. To date, there has been no autopsy report of a patient who died of CFZ-induced cardiotoxicity; thus, in this report, we discuss the pathological findings of such a case.

## 2. Case Presentation

**Figure 1:**
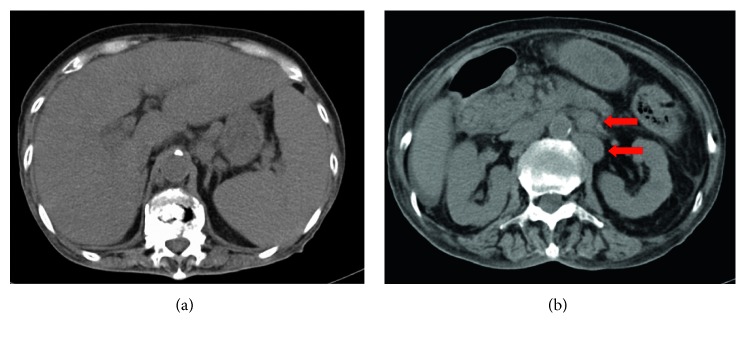
CT image prior to carfilzomib administration. (a) Low uptake throughout the entire liver and splenomegaly could be observed. (b) Para-aortic lymph node swelling was observed (red arrow).

**Figure 2:**
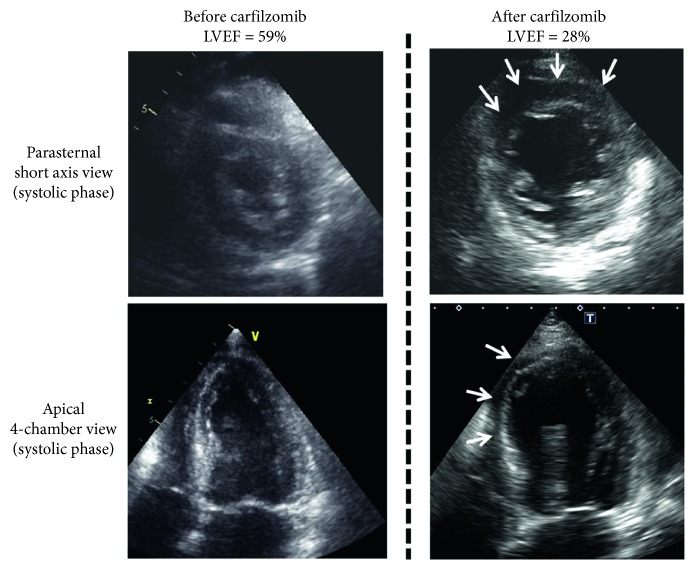
Transthoracic echocardiography beforeand after carfilzomib. After the administration of carfilzomib, the left ventricular ejection fraction decreased to 28% with diffuse wall motion abnormalities, particularly the anteroseptal segment.

In 2016, a 74-year-old woman diagnosed with IgA *κ*-type smoldering MM was hospitalized after developing malaise and fatigability. Physical findings at admission included pale palpebral conjunctiva and edema of the bilateral lower limbs. Laboratory test results were as follows: calcium, 11.4 mg/dL; hemoglobin, 6.1 g/dL; creatinine, 1.54 mg/dL; total protein, 6.4 g/dL; albumin, 3.2 g/dL; IgA, 2923 mg/dL; IgM, 29 mg/dL; IgG, 2253 mg/dL; beta-2-microglobulin, 17.6 mg/L; free kappa light chain, 82.0 mg/L; and free lambda light chain, 18.7 mg/L. Computed tomography (CT) revealed extramedullary tumor in the mediastinum, bilateral axilla, and pulmonary hilum. On fluorescence in situ hybridization, t(14; 16), t(4; 14), t(11; 14), and deletion 17p results were all negative. Symptomatic myeloma (the International Staging System (ISS) stage III and revised ISS stage III) was diagnosed. After high-dose DEX therapy, BOR plus DEX therapy was administered. Although partial response was obtained with four courses, IgA levels had exacerbated by the end of eight courses, and progressive disease was determined. Treatment was switched to LEN (15 mg/day) plus DEX therapy; however, severe cytopenia developed, and treatment was discontinued after one course. CT revealed extramedullary tumor in the para-aortic region, which was thought to indicate disease progression, in addition to low uptake throughout the entire liver (Figure 1). Therefore, treatment was changed to CFZ (20 mg/m^2^ on days 1 and 2; then 36 mg/m^2^ on days 8, 9, 15, and 16) in combination with LEN (5 mg/day) and DEX (KRd) therapy. After administration of KRd therapy, peripheral oxygen saturation had been reduced to 88% early on the next day, and transnasal oxygen therapy was initiated. The brain natriuretic peptide level increased to 418 pg/mL; however, there was no increase in myocardial escape enzymes. Ejection fraction (EF) reduced from 59% to 28% (Figure 2), and radiography revealed pulmonary congestion with pleural effusions. Thus, CFZ-induced acute heart failure was diagnosed. On day 2 of the administration, CFZ was discontinued. Despite the use of norepinephrine, dobutamine, furosemide, and human atrial natriuretic peptide, diuresis was not improved. The patient's respiratory condition deteriorated, and on day 5 following the CFZ administration, noninvasive positive pressure ventilation was initiated. Oxygenation did not improve, and the patient died early morning on day 8 following the CFZ administration. Autopsy was performed following death with the consent of the patient's family.

**Figure 3:**
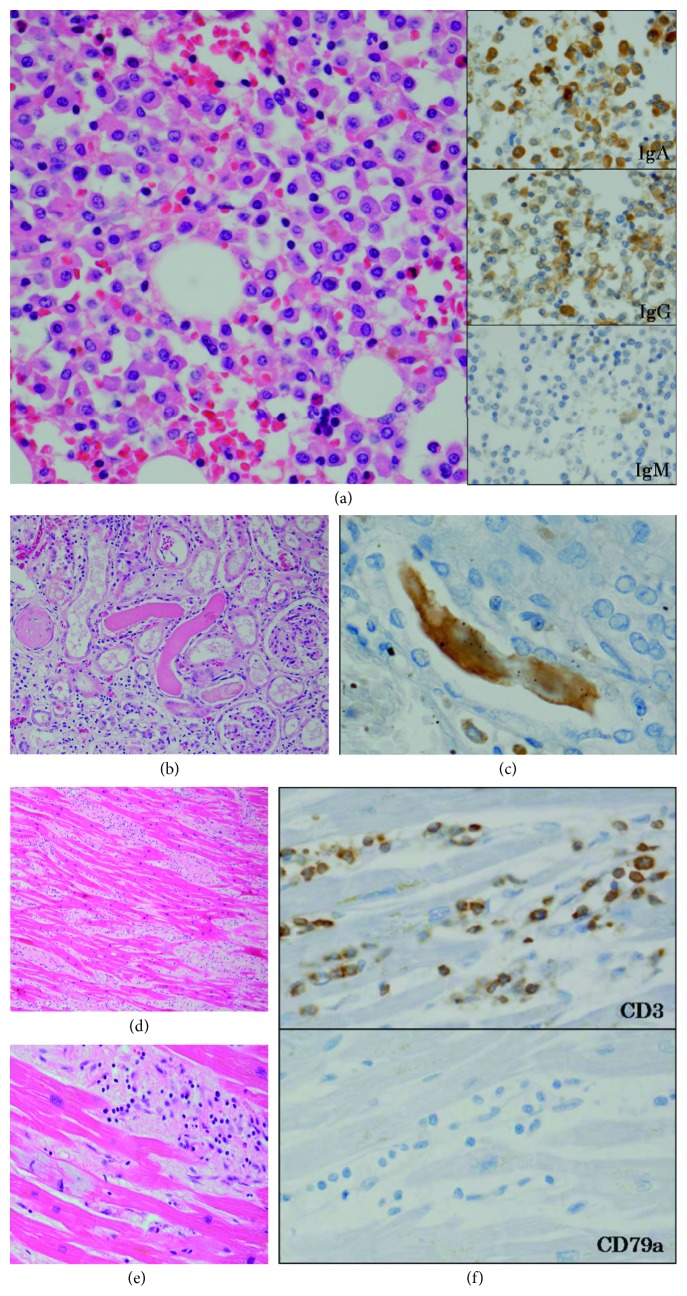
Pathological autopsy findings. (a) Bone marrow contained large basophilic cells with perinuclear halo and proliferation of large cells with distinct nucleoli, which were positive for IgA on immunostaining. (b, c) In the kidneys, the distal convoluted and collecting tubules were filled with eosinophilic amorphous deposits, which was positive for *κ* light chain. (d, e, f) Fibrosis and disarray were observed between the myocardial cells, and these myocardial cells showed basophilic degeneration. Invaded inflammatory cells were positive for CD3 but negative for CD79a.

Macroscopic findings on autopsy revealed multiple vertebral osteolysis, hepatosplenomegaly, renal enlargement, and innumerable lymph node lesions in the paratracheal, tracheal bifurcation, and para-aortic areas. Pathological findings of the bone marrow showed basophilic cells with an eccentric nucleus and proliferation of large cells, which were positive for IgA on immunostaining (Figure 3(a)). The spleen exhibited invasion of similar cells replacing the splenic white pulp, as well as invasion into the splenic red pulp. In the liver, tumor cell invasion was accompanied by destruction of the limiting plates, primarily in the portal region. With the tumor cell invasion, destruction or loss of interlobular ductules and dilation of bile canaliculus were observed. In the kidneys, tumor cell invasion of the interstitial tissue was observed, accompanied by glomerular and renal tubule destruction and loss. The distal convoluted and collecting tubules were filled with eosinophilic amorphous deposits, which was positive for *κ* light chain (Figures 3(b) and 3(c)). There was no obvious thrombus in the coronary artery of the heart, and no infarction was observed in the myocardium. Invasion of inflammatory cells, primarily CD3- (cluster of differentiation 3-) positive lymphocytes, was observed between the myocardial cells, and these myocardial cells showed basophilic degeneration. There was no clear tumor cell invasion or amyloid deposition, and very little myocardial cell necrosis was observed. Between the myocardial cells, fibrosis and disarray were observed (Figures 3(d)–3(f)).

## 3. Discussion

It is well known that CFZ is cardiotoxic, and its clinical use is often associated with cardiovascular events. A recent meta-analysis [4] reported that among 2,594 patients with MM, cardiovascular events of all grades were observed in 18.1% of the patients, whereas events with grade ≥3 in 8.2% of the patients. According to this report, cardiovascular events were divided into four types—heart failure, hypertension, arrhythmias, and ischemic events, with the incidence of the first two being high. While reports of reduced EF following CFZ administration are common, MM is generally considered reversible. In a report from the Mayo Clinic [5], 12 out of 136 patients developed reduced EF following CFZ administration; however, all patients recovered, and the median period until recovery was 2 months. In a different report [6] on 60 patients with MM, 12% experienced reduced EF, which was considered reversible in all of them. Alternatively, Lendvai et al. [7] reported that among 44 patients with MM who received CFZ, six patients developed severely reduced EF, which was irreversible in three of the six cases. Our patient was a rare case, in which reduced EF developed within 24 h of CFZ administration and did not improve but was followed by rapid progression until death. Furthermore, from our search, we found no autopsy reports of death from cardiac hypofunction caused by CFZ.

Several basic studies have addressed the underlying mechanism of CFZ-induced cardiotoxicity; however, the mechanism has not been entirely elucidated. An experiment with rats have reported that exposure to high concentrations of CFZ inhibited chymotrypsin-like proteasomal activity and induced myocardial cell apoptosis [8]. Furthermore, when CFZ is used in combination with doxorubicin, cardiac toxicity is thought to be further exacerbated. An experiment with rabbits has suggested that CFZ has an acetylcholine-mediated vasodilatory effect and increased coronary perfusion pressure by reducing nitroglycerin and nifedipine anticonvulsant activity [9]. It has long been known that high-dose PI downregulates the function of endothelial nitric oxide synthase, a nitric oxide synthesizing enzyme, and can cause vascular endothelial dysfunction [10]. CFZ irreversibly inhibits proteasomes. Compared with other PIs, it may be associated with a higher risk of severe vascular endothelial dysfunction. More recently, however, Efentakis et al. [11] demonstrated that the molecular mechanism of CFZ-induced cardiotoxicity is not related to the inhibition of the proteasome function but to the inhibition of AMPK*α*/mTORC1 pathways derived from increased PP2A activity, which is prevented by metformin.

Major pathological characteristics of the heart in our patient included CD3-positive lymphocyte invasion; however, myocardial cell necrosis was negligible. Myocardial cell disarray can occur in hypertrophic cardiomyopathy and dilated cardiomyopathy, which are the two major types of idiopathic cardiomyopathy. The thickened ventricular muscle and ventricular chamber enlargement, which are characteristics of cardiomyopathy [12], were not observed in our patient, and interstitial fibrosis in our patient was mild. Viral myocarditis is associated with lymphocyte invasion, as in the present case; however, had it been myocarditis, myocardial edematous changes and myocardial cell necrosis would have been remarkable [13], which was not the case in our patient. Sarcoidosis is characterized by epithelioid cell granuloma, which was not observed in our patient. Furthermore, no amyloid deposition was observed, which is a characteristic of cardiac amyloidosis, and no tumor infiltration was observed.

Alternatively, pathological findings of hypersensitivity myocarditis, a drug-induced cardiomyopathy, appeared to agree with this case. Doxorubicin is a drug that commonly causes myocardial impairment; however, the cumulative toxicity of doxorubicin often occurs during a median period of 3 months after administration. By contrast, in fulminant hypersensitivity myocarditis, severe heart failure has been reported to progress during the period from several days to one week following drug administration, and the progress is fatal in some cases [14, 15]. Characteristic histopathological findings of hypersensitivity myocarditis primarily include inflammatory cell invasion, such as lymphocytes in the myocardial stroma, and unremarkable myocardial necrosis [16]. Immunohistologically, cells are primarily CD3-positive lymphocytes, with few CD8-positive T lymphocytes [14]. Lesions have little myocardial cell necrosis; therefore, fibrosis is often unremarkable or mild. These pathological findings are consistent with our patient; however, eosinophilic infiltration, which is a characteristic finding of hypersensitivity myocarditis, was not observed in this case. Thus, it was difficult to make a definitive diagnosis from these pathological findings.

Although no clear relationship was found in a meta-analysis between the incidence of cardiovascular events and age prior to treatment, in the most recent treatment regimen [4], results from 3 phase I/II studies showed that CFZ-induced cardiovascular events are significantly higher in patients older than 75 and that the most important risk factor is hypertension [17]. Recently, the European Myeloma Network in collaboration with the Italian Society of Arterial Hypertension recommended accurate monitoring of blood pressure and early signs and symptoms of cardiac dysfunction in MM patients receiving CFZ [18]. The cardiac function may suddenly decrease as in the present case, although this is rare. Therefore, it is extremely important to carefully monitor the patient's condition for early detection of the onset of cardiovascular events.
